# Establishment of a Dual-Reporter Minigenome System for Respiratory Syncytial Virus

**DOI:** 10.3390/v18030304

**Published:** 2026-02-28

**Authors:** Li Pan, Yunbo Xu, Yihan Ma, Jiaxing Zhang, Chao Wu

**Affiliations:** 1 Institute of Systems and Physical Biology, Shenzhen Bay Laboratory, Shenzhen 518132, China; 2 School of Life Science and Technology, Harbin Institute of Technology, Harbin 150001, China; 3 Shenzhen Medical Academy of Research and Translation (SMART), Shenzhen 518107, China; 4 School of Life Sciences, Westlake University, Hangzhou 310030, China

**Keywords:** respiratory syncytial virus, minigenome, dual-reporter system, antiviral drug evaluation, large polymerase

## Abstract

Respiratory syncytial virus (RSV) poses a significant global health challenge, particularly affecting infants, the elderly, and immunocompromised individuals. Despite recent progress in the development of vaccines and monoclonal antibodies, effective antiviral therapies remain limited. To advance the discovery of antiviral drugs, we have developed a dual-reporter RSV minigenome system, providing a safe and robust platform for antiviral evaluation. This system incorporates NanoLuc luciferase and superfolder GFP (sfGFP) linked by a self-cleaving P2A peptide, allowing for the simultaneous detection of orthogonal signals. Validation with L polymerase inhibitors confirmed the system’s reliability for screening small-molecule inhibitors. The linear correlation observed between the reporter signals enhances the assay’s reliability for antiviral assessment. This dual-reporter minigenome system advances targeted therapeutic strategies against RSV.

## 1. Introduction

Respiratory syncytial virus (RSV) poses a significant global health risk, especially to infants, the elderly, and those with weakened immune systems [1]. It is a leading cause of lower respiratory infections in infants, with millions of cases annually, mainly in resource-limited countries [2,3]. In adults, particularly the elderly, RSV is a major cause of severe respiratory illness [4]; meanwhile, the virus can lead to pneumonia and other serious complications, especially in immunocompromised individuals [5]. There is increasing focus on developing vaccines and antiviral treatments to address RSV’s impact across all age groups [6,7]. Despite extensive research, significant obstacles remain regarding developing RSV interventions. Vaccine candidates struggle to overcome RSV, showing a less than 75% efficacy in elderly trials, limited age-specific use, and incomplete protection against diverse strains [8,9,10]. Similarly, antiviral development remains restricted, with only three FDA-approved agents—the small-molecule inhibitor ribavirin and the monoclonal antibodies palivizumab and nirsevimab—offering limited effectiveness against evolving variants [11]. However, the antiviral drug market is projected to reach $2.5 billion by 2030 [10,12]. Antiviral drug discovery using wild-type or recombinant viruses is technically complex, time-consuming, and requires strict biosafety measures. Minigenome platforms offer a solution without using infectious viral particles, reducing the biosafety risks and speeding up drug discovery [12]. Complementary structure-guided approaches, such as fragment-based screening, have also been used to target conserved nucleoprotein domains implicated in viral RNA synthesis, providing starting points for small-molecule development [13].

The minigenome system is a powerful tool in virology, enabling researchers to dissect viral replication and transcription in cellular contexts without viral infection [14,15]. This system typically employs truncated viral genomic or antigenomic cDNA fragments, in which most viral genes are replaced with reporter genes. This substitution allows for recapitulation of viral processes through the expression of readily detectable markers, such as luciferase or fluorescent proteins [14]. The minigenome platform helps evaluate potential viral RNA synthesis inhibitors [16,17] and identify essential proteins and RNA elements for viral processes, such as genome encapsulation and transcriptional regulation [15,18]. The use of the minigenome system to study filovirus replication and transcription has also been demonstrated [19,20,21]; for example, Ebola virus minigenome assays have been applied to functionally map nucleoprotein determinants and to test inhibitory strategies targeting key protein interfaces [22,23].

RSV gene expression has been examined using several complementary reconstituted systems that differ in how much of the viral life cycle they model. A widely used helper-dependent dicistronic minigenome (e.g., RSV-CAT-LUC) is transfected as in vitro–synthesized RNA into RSV-infected cells, where the infection supplies the viral factors needed for transcription/replication, making it particularly useful for testing how different intergenic regions influence sequential transcription [14]. In contrast, infection-free minigenome assays reconstitute RSV polymerase activity by co-expressing the required viral components alongside a reporter minigenome, enabling the quantitative analysis of transcription (and, depending on the design, the genome replication) without the confounding effects of infection [24]. A more reductionist minimal replication system demonstrated that the functional expression of N, P, and L from cDNA is sufficient to support the replication of RSV genomic RNA analogs, thereby defining the minimal trans-acting requirements for RNA replication [25]. Finally, full-reverse genetics platforms (e.g., BAC-stabilized RSV antigenome cDNA combined with recombination-mediated mutagenesis) allow hypotheses from minigenome assays to be validated in the context of infectious recombinant RSV [26].

Building upon these foundations, we engineered a dual-reporter minigenome system capable of simultaneously expressing NLuc luciferase and sfGFP. This design integrates orthogonal detection modalities into a single platform to ensure robust data reliability. While NLuc offers superior sensitivity and dynamic range for high-throughput biochemical quantification [27], sfGFP provides a critical validation layer through real-time spatial visualization and flow cytometry [28]. Importantly, this dual-readout strategy effectively mitigates false-positive hits, which are common in high-throughput screening—specifically those caused by compounds that directly inhibit luciferase enzymatic activity or stability rather than viral replication—thereby significantly enhancing the fidelity of antiviral drug discovery.

## 2. Materials and Methods

### 2.1. Viruses, Cells, and Plasmids

The RSV A2 strain was obtained from the Chinese Academy of Medical Sciences Collection Center for Pathogenic Microorganisms. The BSR-T7/5 and HEp-2 cell lines were maintained in DMEM supplemented with 10% fetal bovine serum (FBS) at 37 °C in a 5% CO_2_ atmosphere. Furthermore, we constructed expression plasmids that encoded the N, P, M2-1, and L proteins of the RSV A2 strain, specifically pCAGGS(+)-RSV A2-N, pCAGGS(+)-RSV A2-P, pCAGGS(+)-RSV A2-M2-1, and pCAGGS(+)-RSV A2-L. The primers employed for RT-qPCR were as follows: forward primer RGGAATGCTTCACACATTAGT and reverse primer CCTCATTCCTGAGTCTTGCC. We created RSV L active site mutant plasmids named L-D811A, L-G1264A, L-R1339A, and L-G1855S based on published papers or articles [29,30] and confirmed their sequences through sequencing.

### 2.2. Construction of Minigenomes

The reporter gene is flanked by the RSV gene start (GS) and gene end (GE) sequences, which play a crucial role in regulating expression. To more accurately simulate viral replication, non-coding regions (NCs), such as NS1-NC and L-NC, are inserted adjacent to the GS and GE sequences [31]. Furthermore, the minigenome complementary DNA (cDNA) incorporates leader and trailer sequences, along with the T7 promoter, T7 terminator, and ribozymes (e.g., hammerhead ribozyme [HHRz] and hepatitis delta virus ribozyme [HDVRz]) [32,33]. The leader and trailer sequences are responsible for transcription and replication, while the ribozymes ensure precise terminal sequence for both positive and negative strand minigenomes [34]. Transcription from the T7 promoter is facilitated by T7 RNA polymerase (T7 RNP), which is provided through recombinant viruses, expression plasmids, or genetically engineered cell lines [15]. The T7 terminator ensures precise termination of the transcription downstream of the viral genome. To replicate the negative-sense RNA genome of RSV, all non-structural elements, including the T7 promoter, terminator, and ribozymes, were strategically engineered in a reverse complementary orientation. We developed three distinct minigenome constructs: (1) a system that expressed NLuc, (2) a system that expressed sfGFP, and (3) a dual-reporter system that facilitated co-expression of NLuc and sfGFP fused with a self-cleaving peptide P2A.

Design specifications:

(A) Mini-NLuc: 5′-T7 pro→HHRz→trailer→L GE→NC2→NLuc→NC1→NS1 GS→leader→HDVRz→T7 ter;
(B) Mini-sfGFP: 5′-T7 pro→HHRz→trailer→L GE→NC2→sfGFP→NC1→NS1 GS→leader→HDVRz→T7 ter;
(C) Mini-NLuc-sfGFP: 5′-T7 pro→HHRz→trailer→L GE→NC2→sfGFP→P2A→NLuc→NC1→NS1 GS→leader→HDVRz→T7 ter.

A critical feature of the dual-reporter design is the incorporation of the P2A peptide, which facilitates the coordinated translation of both reporter proteins from a single mRNA transcript via ribosomal skipping. The cDNA sequences were synthesized by General Biology (Anhui, China) Co., Ltd., and subsequently cloned into the pOK12 vector to construct the pOK12(−)-Mini-NLuc, pOK12(−)-Mini-sfGFP, and pOK12(−)-Mini-NLuc-sfGFP plasmids. The plasmids were sequenced by BGI Biological Engineering (Shenzhen, China) Co., Ltd.

### 2.3. Transfection System

BSR-T7/5 cells were cultured in 12-well plates and transfected with Lipofectamine 2000 (Thermo Fisher Scientific, Waltham, MA, USA, catalog# 11668019) upon reaching 80–90% confluency. The optimized transfections are detailed in Table 1. At 48 h post-transfection, the sfGFP expression levels were quantified via fluorescence microscopy, while NanoLuc luciferase activity was evaluated using the Nano-Glo^®^ Dual-Luciferase^®^ Reporter Assay System (Promega, US, catalog number N1620), following the manufacturer’s protocol. FLuc was introduced as a transfection control [35]. Because minigenome reporter expression requires co-delivery of the minigenome plasmid together with multiple helper plasmids into the same cell, only a subset of cells is expected to be sfGFP or NLuc positive, which differs from typical single-plasmid transfections that often yield a near-uniform reporter expression.

### 2.4. Small-Molecule Compounds Evaluation

Small-molecule inhibitors of RSV L polymerase, namely, AVG-233 (CAS no. 2151937-80-1) and RSV L-protein-IN-4 (CAS no. 851657-60-8), both sourced from Topscience Co. Ltd., China, were diluted in DMSO to create stock solutions at various concentrations. Cytotoxic effects of these compounds were assessed on BSR T7/5 and HEp-2 cell lines, and the maximum non-toxic dose was determined. Subsequently, BSR-T7/5 cells were cultured and seeded into a 12-well plate. Once the cell confluence reached 80–90%, transfection was conducted using a Lipofectamine 2000 reagent. Six hours post-transfection, the medium was replaced with a fresh one containing different concentrations of small molecules. Samples were incubated at 37 °C in a 5% CO_2_ atmosphere for 48 h. Control groups, which were transfected with minigenome plasmids and treated with DMSO (vehicle control), were assayed for Fluc activity.

### 2.5. Correlation Analysis of NLuc and sfGFP Reporter Signals

Inhibitory effects of AVG-233 and RSV L-protein-IN-4 on the expression of sfGFP and NLuc from Mini-NLuc-sfGFP were tested. An inverted fluorescence microscope was used to observe the sfGFP expression, and the sfGFP fluorescence intensity was analyzed using Fiji (Image J 1.53a) software. Meanwhile, the luciferase activity of treatment groups was determined. Finally, the measured sfGFP fluorescence intensity data and luciferase activity data were fitted with a linear function.

### 2.6. Assessment of Small-Molecule Inhibitors on RSV Replication

HEp-2 cells were cultivated in 12-well plates until 90% confluency. The viral load was calibrated by performing cell counts across triplicate wells. The cells were subsequently infected with RSV A2 (MOI 0.1) for 2 h at 37 °C. Following the adsorption phase, unbound virions were eliminated by performing three washes with serum-free DMEM. Treatment groups were then administered a maintenance medium that contained 2% FBS with different concentrations of AVG-233 or RSV L-protein-IN-4 and incubated for 72 h.

To assess the inhibitory effects of small molecules on RSV A2 replication, immunofluorescence staining was performed. Initially, the cells were washed with PBS to remove the serum and subsequently fixed with 4% paraformaldehyde (Cat# P0099-500mL, Beyotime Biotechnology, Shanghai, China) for 30 min at room temperature. Following three PBS washes, cells were permeabilized with 0.3% Triton X-100 (Cat# P0096-100mL, Beyotime) for 5 min, followed by three additional 5 min PBS washes. Primary antibody staining was conducted using a 1:500 dilution of mouse anti-RSV N monoclonal antibody (Cat# ab94806, Abcam, Cambridge, UK), with incubation at 4 °C for 16 h. After unbound antibodies were removed through three PBS washes, the cells were incubated with a 1:500 dilution of FITC-conjugated goat anti-mouse IgG secondary antibody (Cat# SF131, Solarbio, Beijing, China) at room temperature for 1.5 h, followed by three final PBS washes. Fluorescent signals were acquired using an inverted fluorescence microscope.

To quantitatively evaluate the impact of small molecules on the RSV A2 genome replication, viral genomic RNA was extracted from both the cellular and supernatant fractions of RSV A2-infected cultures treated with different concentrations of AVG-233 and RSV L-protein-IN-4. This extraction was conducted using a commercial viral DNA/RNA extraction kit (Cat#AG21021, Agbio, Changsha, China), following the manufacturer’s guidelines. Subsequently, reverse-transcription quantitative PCR (RT-qPCR) was performed using RSV P gene-specific primers in conjunction with the One Step RT-qPCR Mix (Cat#Q221-01, Vazyme, Nanjing, China). The absolute quantification of viral RNA copies was accomplished using a pOK12-RSV P plasmid standard curve, facilitating a precise assessment of the inhibition effects mediated by the compounds.

### 2.7. Statistical Analysis

Data analysis was performed using GraphPad Prism 9.0 software. Statistical comparisons were made using Student’s two-tailed *t*-test and two-way ANOVA, with significance set at a *p*-value < 0.05.

## 3. Results

### 3.1. Construction and Working Principles of the Dual-Reporter Minigenome System

The RSV minigenome system was developed by substituting the viral coding sequences in the genomic cDNA with reporter genes, such as luciferase or fluorescent proteins. This modification was performed while maintaining essential cis-acting elements, specifically, the leader and trailer sequences, which are crucial for the regulation of transcription and replication.

To develop a multiplexed readout platform, we engineered an advanced dual-reporter minigenome by linking sfGFP and NLuc using a self-cleaving P2A peptide. This configuration facilitates the coordinated expression of both reporters from a single mRNA transcript under a single promoter. The ribosome-skipping activity of the P2A peptide ensures near-complete cleavage of the translated polyprotein, resulting in stoichiometric production of distinct sfGFP and NLuc proteins. The sfGFP reporter was chosen for its remarkable photostability, rapid folding kinetics, and resistance to environmental stressors—characteristics that are essential for sensitive real-time monitoring in complex biological environments. Concurrently, NLuc luciferase offers superior analytical performance due to its compact size (19.1 kDa), high luminescent output (over 100-fold brighter than firefly luciferase), and rapid signal kinetics, facilitating high-temporal-resolution quantification of viral replication dynamics.

**Figure 1:**
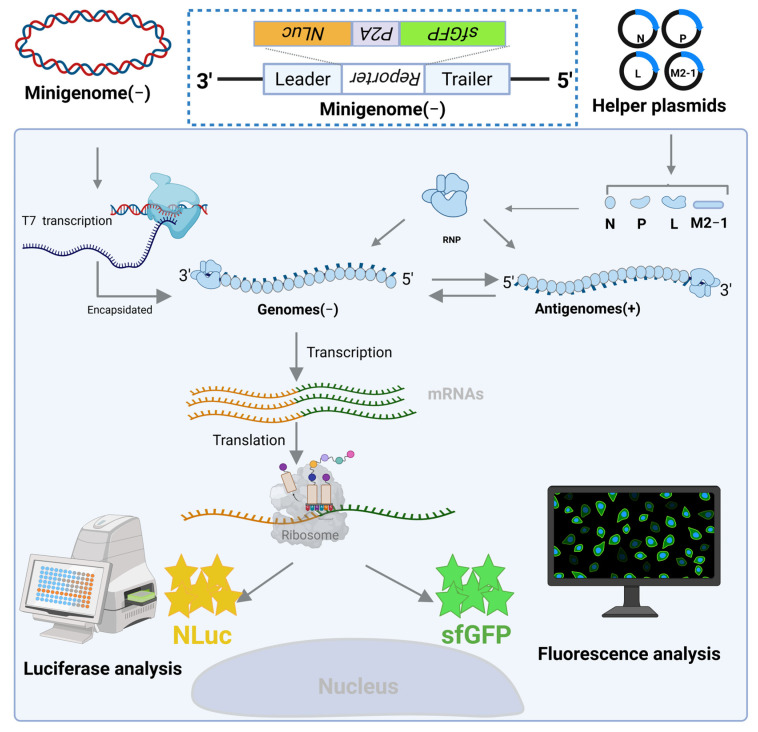
Schematic diagram of the RSV minigenome. The RSV minigenome has been designed as a synthetic analog of the native negative-sense RNA genome, preserving three essential regulatory elements: the leader sequence, which initiates transcription; the trailer sequence, which facilitates genome replication; and a dual-reporter cassette. This cassette incorporates sfGFP and NLuc, separated by P2A peptide, which allows for equimolar co-expression from a single transcriptional unit. *Created in BioRender. Wu, C. (2026) https://BioRender.com/fueytqt*.

The minigenome functions through a specific mechanism: BSR-T7/5 cells, which stably express T7 RNA polymerase, are co-transfected with an engineered minigenome cDNA and helper plasmids encoding the RSV nucleoprotein (N), phosphoprotein (P), large polymerase (L), and transcription elongation factor (M2-1). Post-transfection, the T7-generated minigenome RNA is encapsidated by N to form a functional ribonucleoprotein template. This RNP is subsequently recognized by the polymerase complex (L and P), aided by M2-1, to initiate transcription of mRNA. This mRNA encodes NLuc and sfGFP, linked by a P2A self-cleaving peptide. During translation, ribosomes synthesize a single NLuc-P2A-sfGFP polyprotein, which undergoes autocatalytic cleavage at the P2A site with approximately 98% efficiency, resulting in the production of discrete, functionally active NLuc and sfGFP proteins. Concurrently with the transcription, the polymerase complex facilitates replication by synthesizing antigenomic RNA from the minigenome template, followed by the production of genomic RNA from the antigenomes, thus establishing a self-amplifying replication cycle (Figure 1).

### 3.2. The Mini-NLuc and Mini-sfGFP Systems Exhibit Reporter Activity

**Figure 2:**
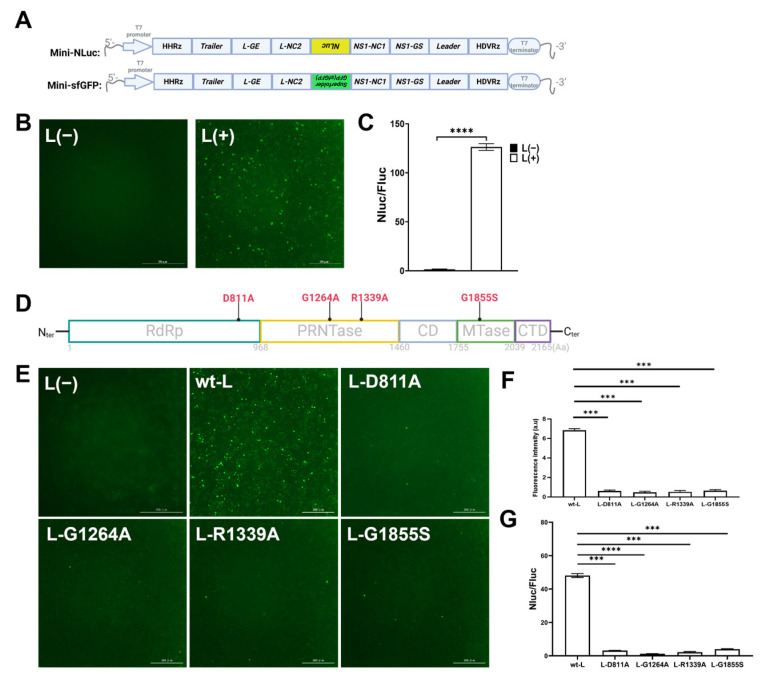
Construction and characterization of the single-reporter gene minigenome. We engineered minigenomes incorporating either NLuc or sfGFP reporter genes to evaluate their functionality through fluorescence and luciferase assays. (**A**) The Mini-NLuc and Mini-sfGFP vectors were structurally identical except for the reporter gene, where Mini-NLuc comprised the T7 promoter, HHRz, RSV trailer, L gene termination sequence, non-coding regions, NLuc sequence, RSV leader, HDVRz, and T7 terminator. Italicized sequences indicate reverse sequences. *Created in BioRender. Wu, C. (2026) https://BioRender.com/sfgj3ee*. (**B**) Co-transfection of Mini-sfGFP with helper plasmids into BSR T7/5 cells resulted in green fluorescence, which was absent without the L protein. Scale bar: 200 μm. (**C**) Co-transfection of Mini-NLuc with helper plasmids into BSR T7/5 cells induces NLuc activity, which was not observed without the L protein. (**D**) Diagram of L mutants. *Created in BioRender. Wu, C. (2026) https://BioRender.com/o1630jk*. (**E**,**F**) Analysis of fluorescent foci and fluorescence intensity of L active site mutants using mini-sfGFP. Scale bar: 300μm. (**G**) NLuc activity evaluation of L active site mutants via mini-NLuc analysis (*** *p* ≤ 0.001; **** *p* < 0.001). Data are presented as the mean ± SD of three independent experiments, each performed in triplicate.

Initially, two minigenome constructs, designated as Mini-NLuc and Mini-sfGFP, were developed in accordance with the experimental design. The Mini-NLuc system incorporated NLuc as its reporter gene, whereas the Mini-sfGFP system utilized sfGFP as the reporter gene (Figure 2A). To evaluate their functionality, either Mini-sfGFP or Mini-NLuc was co-transfected into BSR-T7/5 cells, along with four plasmids that encoded the essential auxiliary proteins of the RNP complex: RSV N, P, L, and M2-1 proteins. These proteins were crucial for the transcription and replication of the minigenome. After 48 h, sfGFP fluorescence was detected via fluorescence microscopy, and the luciferase activity was quantified. The results demonstrated robust green fluorescence in the cells transfected with Mini-sfGFP (Figure 2B) and significant luciferase activity in the cells transfected with Mini-NLuc (Figure 2C), which confirmed efficient reporter gene expression facilitated by the helper protein complex. Control experiments underscored the critical role of the RSV L protein, as its absence resulted in neither sfGFP fluorescence nor luciferase activity.

Subsequently, the engineered L mutants were assessed utilizing mini-NLuc and mini-sfGFP assays (Figure 2D). Our findings indicate that in comparison with the wild-type L, the sfGFP fluorescent foci associated with the L active site mutants exhibited a significant reduction (Figure 2E,F), while the NLuc activity was also markedly diminished (Figure 2G).

### 3.3. Mini-NLuc-sfGFP Co-Expresses sfGFP and NLuc

**Figure 3:**
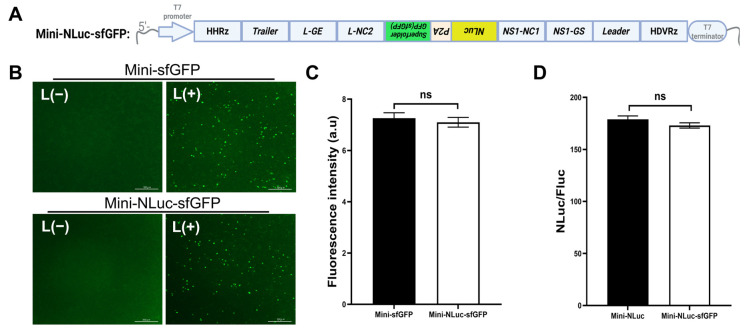
Construction and characterization of Mini-NLuc-sfGFP. (**A**) Mini-NLuc-sfGFP’s structure included the T7 promoter, HHRz sequence, trailer, L gene end signal and untranslated regions, NLuc sequence, NS1 gene untranslated and gen start signal, leader region, HDVRz sequence, and T7 terminator. Italicized sequences indicate reverse sequences. *Created in BioRender. Wu, C. (2026) https://BioRender.com/sfgj3ee*. (**B**) Co-transfection with helper plasmids in BSR-T7/5 cells produced green fluorescent spots, which were absent without L. Scale bar: 300 μm. (**C**) sfGFP fluorescence in Mini-NLuc-sfGFP-transfected cells matched that of Mini-sfGFP-transfected cells. (**D**) Luciferase activity was present in the cells co-transfected with Mini-NLuc-sfGFP and helper plasmids, but absent without the L protein. Data are presented as the mean ± SD of three independent experiments, each performed in triplicate.

We engineered the dual-reporter minigenome, Mini-NLuc-sfGFP, which integrated the benefits of Mini-NLuc and Mini-sfGFP. This construct facilitated the concurrent expression of sfGFP and NLuc through a P2A peptide linker (Figure 3A). For the functional evaluation, Mini-NLuc-sfGFP was co-transfected with plasmids that encoded the RSV N, P, L, and M2-1 proteins into BSR-T7/5 cells. At 48 h post-transfection, the sfGFP fluorescence and NLuc activity were assessed. The results indicated that the sfGFP expression in Mini-NLuc-sfGFP-transfected cells was comparable with that in the Mini-sfGFP-transfected cells (Figure 3B,C), and the NLuc activity was analogous to that observed in Mini-NLuc-transfected cells (Figure 3D). These findings confirm that Mini-NLuc-sfGFP successfully integrates the functionalities of both single-reporter systems, facilitating efficient co-expression of sfGFP and NLuc without compromising the performance of either reporter.

### 3.4. Mini-NLuc-sfGFP Is an Efficient Tool for Evaluating Antiviral Small Molecules

**Figure 4:**
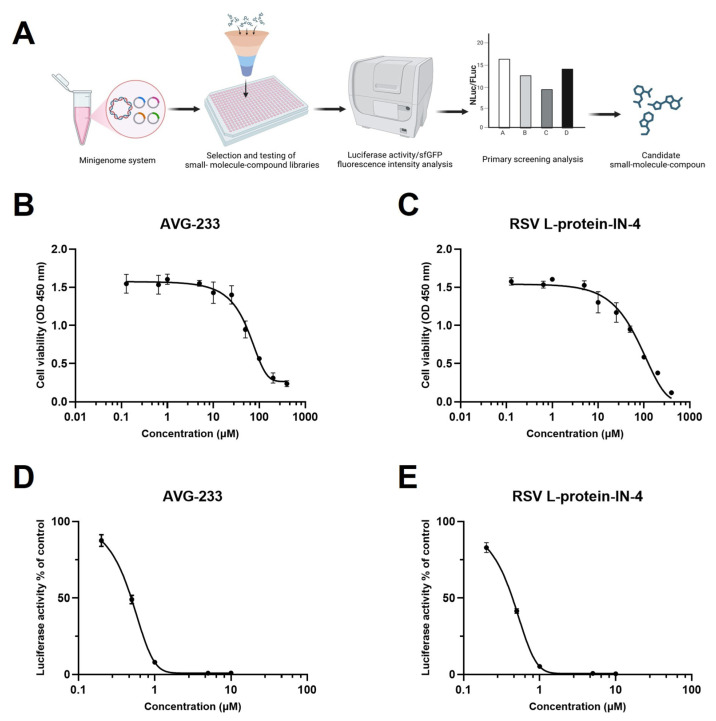
Mini-NLuc-sfGFP for small-molecule evaluation. (**A**) The experimental protocol involved transfecting the Mini-NLuc-sfGFP minigenome into BSR-T7/5 cells, followed by treatment with a range of small molecules. The inhibitory effects were initially assessed by quantifying the fluorescence intensity or NLuc activity, which led to the selection of promising candidates for further investigation. *Created in BioRender. Wu, C. (2026) https://BioRender.com/8qct7m8*. (**B**) AVG-233 reduced the BSR-T7/5 cell viability in a concentration-dependent manner (OD_450_). (**C**) Similarly, RSV L-protein-IN-4 reduced the BSR-T7/5 cell viability in a concentration-dependent manner (OD_450_). (**D**) AVG-233 demonstrated a dose-dependent inhibition of Mini-NLuc-sfGFP reporter gene expression, as indicated by the luciferase activity. (**E**) Similarly, RSV L-protein-IN-4 exhibited the dose-dependent inhibition of the Mini-NLuc-sfGFP reporter gene expression, also measured using the luciferase activity. Data are presented as the mean ± SD of three independent experiments, each performed in triplicate.

The Mini-NLuc-sfGFP system was employed to evaluate small molecules for antiviral activity against RSV, with AVG-233 and RSV L protein-IN-4 selected as the test compounds (Figure 4A). Initially, BSR-T7/5 cells were co-transfected with plasmids encoding the RSV proteins N, P, L, and M2-1. Subsequently, AVG-233 and RSV L protein-IN-4 were added individually, and after a 48 h incubation period, their capacity to inhibit minigenome expression was assessed by monitoring the sfGFP fluorescence intensity and measuring the NLuc activity. The results indicated that both compounds significantly reduced the NLuc activity in a dose-dependent manner (Figure 4B,C), underscoring the practical utility of this system for preliminary antiviral evaluation. Based on these findings, subsequent experiments will employ lower concentrations of the compounds to further explore their inhibitory effects on reporter gene signals.

### 3.5. Mini-NLuc-sfGFP Shows a Linear Correlation Between NLuc and sfGFP Signals

**Figure 5:**
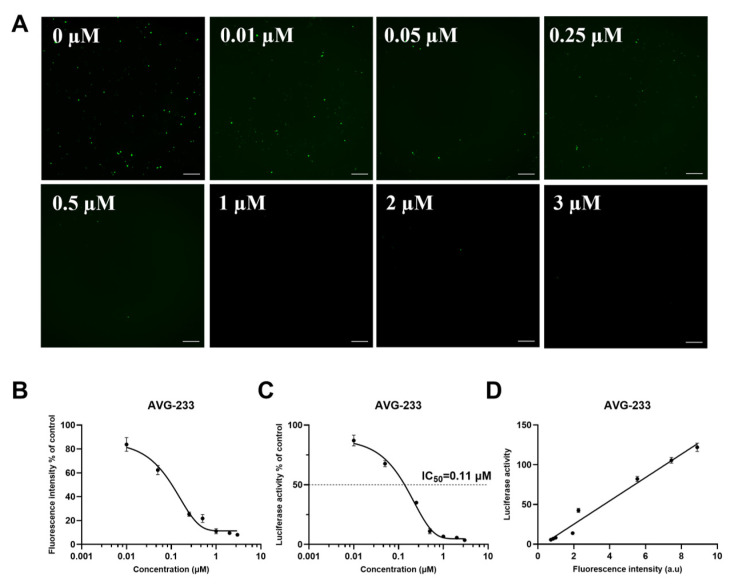
Analysis of the linear relationship between sfGFP and NLuc in Mini-NLuc-sfGFP to evaluate AVG-233. (**A**) Effects of AVG-233 on the sfGFP fluorescence at 0, 0.01, 0.05, 0.25, 0.5, 1, 2, and 3 μM. (**B**,**C**) AVG-233 demonstrated a dose-dependent inhibition of Mini-NLuc-sfGFP reporter gene expression, as indicated by the fluorescence intensity and luciferase activity. (**D**) Linear regression showed a significant correlation between sfGFP and NLuc. Scale bar: 400 μm. Data are presented as the mean ± SD of three independent experiments, each performed in triplicate.

**Figure 6:**
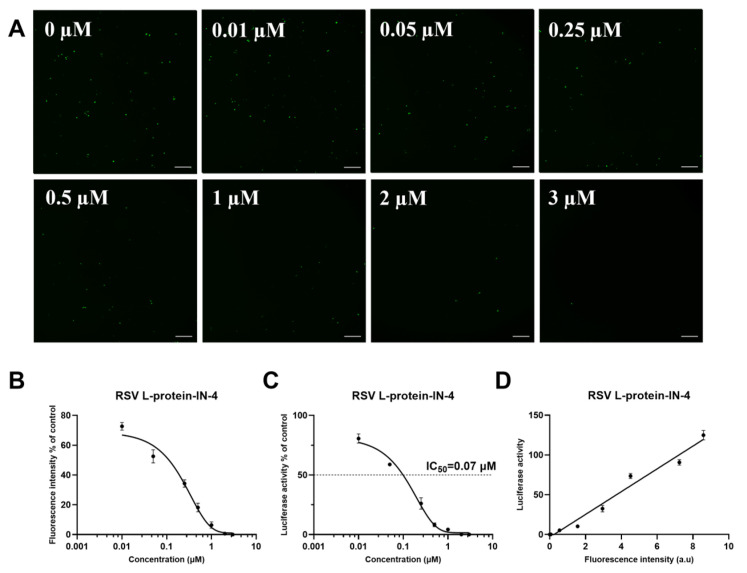
Analysis of the linear relationship between sfGFP and NLuc in Mini-NLuc-sfGFP to evaluate RSV L-protein-IN-4. (**A**) Effects of RSV L-protein-IN-4 on the sfGFP fluorescence at 0, 0.01, 0.05, 0.25, 0.5, 1, 2, and 3 μM. (**B**,**C**) RSV L-protein-IN-4 demonstrated a dose-dependent inhibition of Mini-NLuc-sfGFP reporter gene expression, as indicated by fluorescence intensity and luciferase activity. (**D**) Linear regression showed a significant correlation between sfGFP and NLuc. Scale bar: 400 μm. Data are presented as the mean ± SD of three independent experiments, each performed in triplicate.

We assessed the linear relationship between the sfGFP fluorescence and NLuc activity in the Mini-NLuc-sfGFP system by examining the effects of two RSV L protein inhibitors: AVG-233 and RSV L-protein-IN-4. Both inhibitors were tested across a range of concentrations (the compound concentrations used in the minigenome were selected based on the viability assays shown in Figure 4B,C to minimize potential cytotoxicity), with measurements taken for sfGFP fluorescence and NLuc activity. The results indicated that AVG-233 induced dose-dependent reductions in the sfGFP fluorescence (Figure 5A,B) and NLuc activity (Figure 5C), demonstrating a strong linear correlation (R^2^ = 0.977, Figure 5D). Similarly, RSV L-protein-IN-4 led to dose-dependent decreases in the sfGFP fluorescence (Figure 6A,B) and NLuc activity (Figure 6C), also exhibiting a strong linear correlation (R^2^ = 0.974, Figure 6D). In conclusion, the Mini-NLuc-sfGFP system effectively produced concurrent sfGFP and NLuc signals with a robust linear relationship, underscoring its reliability for screening antiviral compounds against RSV and supporting future drug development endeavors.

### 3.6. Mini-NLuc-sfGFP Strongly Correlates with Viral Inhibition in Small-Molecule Evaluations

To validate the Mini-NLuc-sfGFP system for assessing small-molecule inhibitors of RSV replication, we evaluated two compounds—AVG-233 and RSV L-protein-IN-4—which are both known to target the RSV L protein. Their inhibitory effects across concentrations were analyzed using indirect immunofluorescence assays (IFAs) and reverse-transcription quantitative PCR (RT-qPCR).

Under our RSV A2 infection conditions (MOI 0.1, 72 h post infection), typical RSV-induced cytopathic effects (CPEs) were observed. Infected cells gradually aggregated and formed large syncytia, and at later stages, these syncytia were accompanied by evident cell rounding and occasional cell death/detachment. Therefore, all downstream readouts (immunofluorescence staining and RT–qPCR) were collected at 72 h post infection, a defined time window in which the CPEs were apparent but the monolayer remained sufficiently intact and adherent to allow reliable fixation, staining, and quantitative analysis.

**Figure 7:**
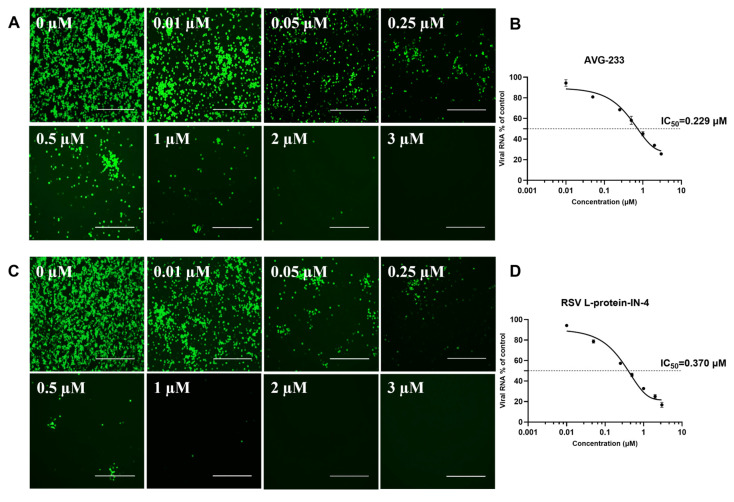
Validation of small-molecule inhibitory effects assessed by Mini-NLuc-sfGFP and confirmed in RSV A2. RSV A2 infection was conducted at a multiplicity of infection (MOI) of 0.1, utilizing a primary antibody of mouse anti-RSV F and a secondary antibody of goat anti-mouse FITC conjugate. (**A**) Immunofluorescence imaging showed that AVG-233 inhibited viral infection at 0, 0.01, 0.05, 0.25, 0.5, 1, 2, and 3 μM. (**B**) Viral genome copy numbers via RT-qPCR at equivalent concentrations. (**C**) Immunofluorescence imaging showed that RSV L-protein-IN-4 inhibited viral infection at 0, 0.01, 0.05, 0.25, 0.5, 1, 2, and 3 μM. (**D**) Viral genome copy numbers via RT-qPCR at equivalent concentrations. Scale bar: 400 μm. Data are presented as the mean ± SD of three independent experiments, each performed in triplicate.

The results show that increasing concentrations of AVG-233 (Figure 7A) and RSV L-protein-IN-4 (Figure 7C) reduced the viral infection foci. RT-qPCR further confirmed dose-dependent suppression of the wild-type RSV A2 genome replication: AVG-233 reduced viral genome copies from 0 to 3 μM (Figure 7C), whereas RSV L-protein-IN-4 inhibited replication within its safe concentration range (Figure 7D).

The integrations of IFA and RT-qPCR facilitated a thorough examination of the antiviral efficacy and dose-dependency of the compounds, thereby affirming a robust correlation between the Mini-NLuc-sfGFP system and the live virus.

## 4. Discussion

RSV affects people of all ages, with children, the elderly, and the immunocompromised being most at risk [36]. Although there are no global antiviral treatments for RSV, the FDA approved two vaccines in 2023 [37,38]. However, the FDA and CDC have warned that these vaccines might increase the risk of Guillain–Barré syndrome in older adults [39,40]. RSV small–molecule inhibitors show promise as targeted therapies for high-risk groups, such as newborns, the elderly, and those with weakened immune systems, including cancer patients and organ transplant recipients [41,42]. After the zero-COVID policy was optimized in late 2022, RSV cases surged in Northern China, with the winter 2023 peak reaching a nine-year high [43]. This underscores the ongoing challenges in managing RSV and the urgent need for effective treatments.

Traditional RSV minigenome systems rely on BSR T7/5 cells or recombinant vaccinia viruses to provide T7 RNA polymerase. However, vaccinia-based methods can be unstable and introduce interfering viral components. In contrast, the Mini-NLuc-sfGFP system works efficiently in BSR T7/5 cells, which already express T7 RNA polymerase [44]. This system, which integrates fluorescence and luciferase reporters, facilitates the qualitative and quantitative evaluation of small molecules, thereby supporting drug screening and efficacy assessment. Notably, related filovirus minigenome reporter assays have been implemented in a 96-well, internally normalized dual-luciferase form, illustrating the feasibility of scalable reporter-based testing [45]. Beyond its application in drug discovery, the dual-reporter minigenome serves as a crucial tool for investigating viral protein functions and replication, thereby advancing our comprehension of RSV biology and aiding in the development of antiviral strategies [46]. Additionally, it forecasts the success of recombinant virus rescue, thereby enhancing experimental outcomes and timelines [26,27]. As demonstrated in Figure 2, L mutants led to a marked decrease in reporter signals. The application of this minigenome system permits an initial evaluation of the impact of key RSV gene mutations on protein function. This methodology not only conserves time by obviating the need for recombinant virus construction but also contributes to the targeted development of inhibitors. The system’s strong compatibility and linearity of reporter signals improve accuracy and reliability. A P2A sequence between sfGFP and NLuc genes enables efficient co-expression from one mRNA, enhancing system efficiency. We chose sfGFP and NLuc for their brightness, rapid folding, stability, low background fluorescence, and quick response, making them ideal for early signal detection and sensitive experiments. This dual-reporter system provides reliable results, even under challenging conditions, such as temperature or pH fluctuations and toxin exposure, ensuring consistent reporter gene expression [47,48].

RSV depends on its polymerase complex (N, P, L, and M2-1 proteins) for genome replication [49]. The multifunctional L polymerase is crucial for transcribing viral mRNA and synthesizing complementary RNA, making it a key target for RSV antiviral development [50,51,52]. Our study used the RSV minigenome system to evaluate two small molecules, AVG-233 and RSV L-protein-IN-4, targeting the L polymerase. AVG-233 non-competitively inhibits RNA synthesis post-initiation, while RSV L-protein-IN-4 blocks mRNA capping, inhibiting mRNA synthesis and viral replication [53,54]. Both compounds dose-dependently reduced sfGFP and NLuc reporter signals, as confirmed in wild-type RSV A2 virus assays, demonstrating the Mini-NLuc-sfGFP system’s effectiveness in evaluating RSV-targeting small molecules.

The Mini-NLuc-sfGFP system is used not only to discover drugs that target the L polymerase but also to investigate RSV’s replication and transcription mechanisms. This includes studying the leader and trailer regions’ roles in genome replication and the effects of viral proteins such as N, P, L, and M2-1 on replication compartment formation. Increased N protein levels boost viral RNA replication, while M2-1 does not affect RNA replication or mRNA to antigenome synthesis ratios [55]. Additionally, research on related viruses, such as Ebola, has utilized minigenomes to characterize RNA editing mechanisms and identify essential cis-acting sequences near the editing site [56]. Another study highlighted that specific basic residues, particularly in the first basic patch, are vital for viral RNA synthesis and replication complex formation, influencing VP35-nucleoprotein interactions [57]. These findings indicate that our mini-NLuc-sfGFP system can enhance understanding of RSV replication and transcription mechanisms.

The Mini-NLuc-sfGFP system offers significant benefits but also has limitations and areas for improvement. It is optimized for a stable T7 polymerase-expressing cell line, removing the need for external polymerase delivery, but this limits its use to certain cell types, excluding those relevant to RSV, such as human airway epithelial cultures. Future improvements will require alternative polymerase delivery methods. Furthermore, as the co-transfection efficiency remains a primary bottleneck inherent to multi-plasmid minigenome systems, our ongoing efforts will focus on the further optimization of this platform. We aim to progressively miniaturize the assay for 96- and 384-well formats and rigorously evaluate the Z’ factor, thereby establishing robust data validation for high-throughput screening (HTS) applications. The system’s main advantage over single-reporter systems is its built-in internal validation: sfGFP provides quick, spatial, and qualitative feedback on transfection efficiency and minigenome activity, aiding in visual assessment and troubleshooting, while NLuc delivers sensitive, quantitative data ideal for dose–response assays and high-throughput screening.

For most applications, it is advisable to routinely measure both reporters: (1) In the context of drug screening and small-molecule evaluation, NLuc should serve as the primary quantitative readout due to its superior sensitivity, extensive dynamic range, and minimal background interference. The sfGFP signal plays a critical role in quality control, ensuring that any observed reduction in luminescence is attributable to authentic antiviral activity rather than compound cytotoxicity or inadequate transfection, both of which could also lead to diminished GFP fluorescence. We recommend employing sfGFP for rapid primary screening, such as high-throughput visual inspections to eliminate inactive compounds, thereby reducing the number of samples necessitating NLuc quantification. Subsequently, NLuc should be utilized to generate precise IC_50_ data for identified hit compounds. For long-term assays exceeding 48 h, it is advisable to prioritize NLuc, as sfGFP may be susceptible to photobleaching or accumulation in lysosomes, which could result in signal saturation. (2) In investigations of viral replication and transcription mechanisms, sfGFP is invaluable for monitoring the temporal and spatial dynamics of minigenome activity in live cells, while NLuc offers precise kinetic data derived from the same sample lysates. A divergence in the signals from these two reporters should not be interpreted as indicative of system failure; rather, it presents an opportunity for more in-depth analysis. A notable reduction in the NLuc signal, accompanied by a stable sfGFP fluorescence, may imply a specific inhibition of translation or a potential issue with the luciferase assay itself, such as reagent instability. Conversely, a simultaneous loss of both signals strongly suggests a defect in upstream processes, such as RNA synthesis or overall minigenome integrity. Consequently, the dual-reporter system not only enhances the reliability of the data but also provides a more comprehensive toolkit for diagnosing the mechanisms of action of antiviral compounds or the functional consequences of mutations in viral proteins.

Considering these factors, the Mini-NLuc-sfGFP system offers a robust platform for RSV research, supporting both antiviral discovery and fundamental virology. By expanding existing RSV minigenome tools with dual, complementary readouts, it enables efficient evaluation of antiviral candidates and functional analysis of key viral genes.

## Figures and Tables

**Table 1:** Minigenome transfection protocol.

Groups	Shared Plasmids	Additional Plasmids
I	pCAGGS(+)-RSV A2-N (0.5 µg) pCAGGS(+)-RSV A2-P (0.5 µg) pCAGGS(+)-RSV A2-M2-1 (0.5 µg) pcDNA3.1(+)-FLuc (0.1 µg)	pOK12(−)-Mini-NLuc-sfGFP (1 µg) +pCAGGS(+)-RSV A2-L (1 µg)
II		pOK12(−)-Mini-NLuc-sfGFP (1 µg)
III		pOK12(−)--NLuc (1 µg) +pCAGGS(+)-RSV A2-L (1 µg)
IV		pOK12(−)-Mini-NLuc (1 µg)
V		pOK12(−)-Mini-sfGFP (1 µg) +pCAGGS(+)-RSV A2-L (1 µg)
VI		pOK12(−)-Mini-sfGFP (1 µg)

## Data Availability

The original contributions presented in this study are included in the article Further inquiries can be directed to the corresponding author.
